# Perception-Action Integration Is Modulated by the Catecholaminergic System Depending on Learning Experience

**DOI:** 10.1093/ijnp/pyab012

**Published:** 2021-03-17

**Authors:** Elena Eggert, Annet Bluschke, Adam Takacs, Maximilian Kleimaker, Alexander Münchau, Veit Roessner, Moritz Mückschel, Christian Beste

**Affiliations:** 1 Cognitive Neurophysiology, Department of Child and Adolescent Psychiatry, Faculty of Medicine, TU Dresden, Germany; 2 Institute of Systems Motor Science, University of Lübeck, Germany

**Keywords:** Catecholaminergic system, methylphenidate, perception, action, theory of event coding

## Abstract

**Background:**

The process underlying the integration of perception and action is a focal topic in neuroscientific research and cognitive frameworks such as the theory of event coding have been developed to explain the mechanisms of perception-action integration. The neurobiological underpinnings are poorly understood. While it has been suggested that the catecholaminergic system may play a role, there are opposing predictions regarding the effects of catecholamines on perception-action integration.

**Methods:**

Methylphenidate (MPH) is a compound commonly used to modulate the catecholaminergic system. In a double-blind, randomized crossover study design, we examined the effect of MPH (0.25 mg/kg) on perception-action integration using an established “event file coding” paradigm in a group of n = 45 healthy young adults.

**Results:**

The data reveal that, compared with the placebo, MPH attenuates binding effects based on the established associations between stimuli and responses, provided participants are already familiar with the task. However, without prior task experience, MPH did not modulate performance compared with the placebo.

**Conclusions:**

Catecholamines and learning experience interactively modulate perception-action integration, especially when perception-action associations have to be reconfigured. The data suggest there is a gain control–based mechanism underlying the interactive effects of learning/task experience and catecholaminergic activity during perception-action integration.

**Significance Statement**

The integration of perception and action is central to goal-directed behavior, but little is known regarding the relevant neurobiological systems. We modulated the catecholaminergic system with methylphenidate (MPH), a first-line drug for the treatment of neuropsychiatric disorders, particularly attention deficit hyperactivity disorder (ADHD), where difficulties in perception-action integration have been described. We demonstrate that the catecholaminergic system plays a central role in perception-action integration. However, pharmacological attempts to enhance perception-action integration (e.g., in psychiatric populations) must consider the role of prior learning and task experience. The study establishes a close link between learning and catecholaminergic effects in the context of the integration of perceptions with appropriate actions, demonstrating findings of considerable theoretical and clinical relevance.

## Introduction

One of the foci of cognitive neuroscience research has been the investigation of processes underlying how sensory information and corresponding actions manifest as mental representations and guide behavior. For example, when your cell phone rings, you are likely to pick up the phone. When your friend’s cell phone rings with the same tone, you may, at least for a few moments, be alerted to pick up a phone but then refrain from doing so when you notice that it is not yours. This example illustrates how strongly associations (bindings) between stimuli and responses can determine behavior. A prominent conceptual framework detailing how perception and action are integrated is the theory of event coding (Hommel, 1998; Hommel et al., 2001). It postulates that sensory stimuli and actions are not processed and stored separately but rather share a common coding mechanism (Hommel, 2009). Thus, sensory stimuli are represented by “object files,” which include information regarding the features of, for example, a visual object (such as location, color, shape) and sensory feedback of the response to the stimulus. Accordingly, actions are represented by “action files” comprising information regarding specific aspects of the response to a stimulus as well as respective perceptual consequences (e.g., which finger to use, etc.). Object and action files are bound together in “event files” representing multi-layered networks containing information concerning stimulus-response (S-R) associations. Once established, the activation of an event file can be induced by the perception of the associated stimulus and then spread to the other representations inherent to the network (Hommel, 2009). Due to the robustness of event file bindings, previously established bindings strongly affect the efficacy of subsequent responses (Colzato et al., 2006; Hydock et al., 2013; Petruo et al., 2019): Responses are facilitated when 2 consecutive stimuli display a high level of feature overlap and thus a reactivation of the same response is required. Yet, event file bindings may impair the response selection process when similar stimuli require different responses, as illustrated in the introductory example. These findings are well-documented and have been referred to as partial repetition benefits and costs, respectively (Hommel, 2004, 2009; Colzato et al., 2006). In recent years, further frameworks regarding action control and perception-action integration have been developed (Schmidt et al., 2016; Hommel, 2019; Frings et al., 2020), stressing that not only binding processes between stimulus and response feature are central for event file coding processes, but also retrieval processes. In the current study, however, we relied on a more classical experimental approach to examine event file binding in which the contribution of retrieval processes can hardly be examined in detail. We do so because for this experimental approach there is good evidence from previous studies including genetic association analyses and neurological disorders that catecholaminergic (i.e., dopaminergic) activity has an impact on event file coding (Colzato and Hommel, 2008; Colzato et al., 2012, 2013). However, the neuropharmacological underpinnings of the catecholaminergic system in event file coding are still poorly understood.

The catecholaminergic system, which encompasses dopamine (DA) and norepinephrine (NE) activity. The catecholaminergic system plays a central role in the stability of information in working memory (Seamans and Yang, 2004; Robbins and Arnsten, 2009; Arnsten, 2011; Cools and D’Esposito, 2011), and studies indicate that increased levels of catecholamines enhance the strength of working memory representations (Kentros et al., 2004; Hamilton et al., 2010). Since event files can be considered representations of S-R bindings in episodic (working) memory traces (Hommel, 2009; Takacs et al., 2020a), it is possible that modulations of the catecholaminergic system may affect event file processing. A compound commonly used for the modulation of catecholaminergic activity is methylphenidate (MPH), which is also the recommended first-line treatment of attention-deficit hyperactiviy disorder (American Academy of Pediatrics, 2011; Childress and Sallee, 2014; Mattingly et al., 2017). Since MPH increases postsynaptic levels of DA/NE by blocking transporters that mediate DA/NE re-uptake (Faraone, 2018), it may be hypothesized that increased catecholaminergic concentrations after MPH intake may strengthen representations of event file bindings. Crucially, this could impair performance whenever identical/similar stimuli require different responses or when the same response must be executed using a different stimulus input. In these cases, previously established bindings in the event file have to be reconfigured, which is more demanding when event file bindings are highly stable (Hommel, 2009). Therefore, one possible hypothesis of the current study is that MPH impairs performance whenever identical or similar stimuli require a different response, or when the same response must be executed using a different stimulus input.

However, there is mounting evidence that the effects of MPH depend on the level of prior experience or familiarity with a given task (Bensmann et al., 2019; Mückschel et al., 2020a, 2020b). These insights have been gained through the use of cross-over study designs. The finding that MPH effects depend on prior task experience/learning is also relevant in the context of event file processing since learning can modulate event file coding (Eberhardt et al., 2017) and event files can be seen as episodic (working) memory traces (Hommel, 2009; Takacs et al., 2020a). It has been suggested that the interplay of prior task experience and MPH is based on so-called gain control principles (Bensmann et al., 2019). High gain control equals a high signal-to-noise ratio in neural circuits (Cohen et al., 2002; Winterer and Weinberger, 2004; Rolls et al., 2008; Kroener et al., 2009; Vander Weele et al., 2018) and thus a high ability to efficiently process input signals (Pertermann et al., 2019) and select responses (Wolff et al., 2017, 2018; Adelhöfer et al., 2018, 2019). Since gain control mechanisms also underlie learning and plasticity (Dosher and Lu, 1998; Gold et al., 1999) and evidence suggests that both the DA and the NE system modulate gain control mechanisms (Hauser et al., 2016), it seems reasonable to assume that the interplay of prior task experience and MPH effects is based on gain control principles (Bensmann et al., 2019). The fact that high gain control leads to a more efficient processing of input signals and enhances response selection processes affects the hypotheses regarding MPH/learning effects on event file binding and perception-action integration. Efficient response selection processes are particularly important when it is necessary to reconfigure strong bindings in an event file (Hommel, 2009), that is, whenever identical/similar stimuli require different responses or when the same response must be executed using different stimulus input. The consideration of gain control mechanisms possibly being modulated by MPH leads to a prediction contrary to the hypothesis described above. Based on gain control mechanisms, it could be hypothesized that MPH administration enhances performance whenever identical/similar stimuli require different response or when the same response must be executed using a different stimulus input.

In the current study, we tested these opposing hypotheses regarding the relevance of the catecholaminergic system for perception-action integration (event file binding). We did so in a randomized, double-blind cross-over design in which we administered MPH at a dose of 0.25 mg/kg body weight.

## Materials and Methods

### Participants and a Priori Power Analysis

The sample consisted of n = 45 healthy participants between 20 and 30 years of age (19 females; mean age 23.6, SD = 2.52). The sample size was comparable to previous studies with equivalent study designs (Bensmann et al., 2019; Mückschel et al., 2020a, 2020b). A sensitivity analysis was conducted with G*Power (Faul et al., 2007) to determine the detectable effect size. Based on a sample size of n* = *45 and a power of .95, the analysis revealed that a small effect size of *f* = .140, equivalent to ƞ ^2^ = .019, could be detected. Therefore, obtained effects larger than ƞ ^2^ = .019 can be regarded as reliable. Prior to the sessions, all participants filled in the Adult Self-Report for ages 18–59 years in the form of an online questionnaire aimed at screening for signs of psychiatric problems and difficulties in adaptive functioning. Only participants without any history of psychiatric or neurological disorders and who were not regularly taking any medication were included in the study. Only women currently taking oral contraceptives were eligible to take part in the study. All participants signed an informed consent form and received either a financial reimbursement or course credit for their participation. The study was approved by the ethics committee of the Faculty of Medicine of the TU Dresden.

### MPH Administration

All participants took part in 2 sessions. Using a randomized, double-blind cross-over design, they were randomly allocated to 1 of 2 groups: 1 group received the MPH during the first session (and an identical-looking placebo during the second session; n = 23), while the other group received the placebo during the first session (and MPH during the second session; n = 22). Thereby, it was possible to examine the interaction between MPH administration and the effects of prior learning experience. Both the participants and the researchers were blind to the order of substance administration. Participants were given a single dose of immediate-release MPH (0.25 mg/kg body weight). We chose a single dose of 0.25 mg/kg MPH because previous studies by our group consistently revealed interaction effects with prior learning experience in various cognitive functions using these MPH dosages (Bensmann et al., 2019; Mückschel et al., 2020a, 2020b). As in these studies, experimental testing in the present study started approximately 75 minutes after MPH intake. Accordingly, the task was completed within the time period in which MPH plasma levels are estimated to peak (Challman and Lipsky, 2000; Rösler et al., 2009). The interval between sessions ranged from a minimum of 24 hours to a maximum of 14 days (mean interval 5.24 days ± 3.09).

### Task

**Figure 1.**
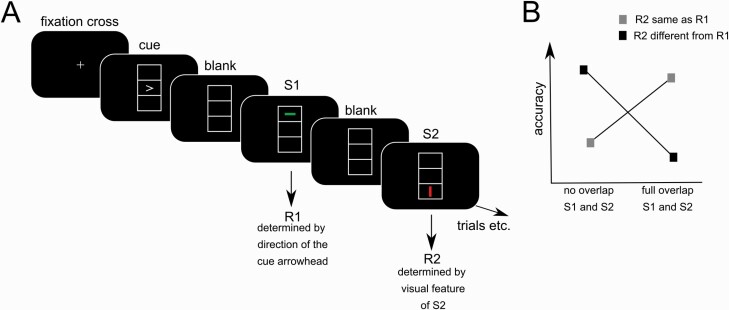
(A) The stimuli sequence of the experimental paradigm. For details regarding the timing, please refer to the Methods section. (B) Schematic illustration (not based on real data) of how event file binding is reflected on a statistical level. On a statistical level, binding is indicated by a significant interaction of “feature overlap” and “response.” Depending on “response,” variations in the degree of stimulus feature overlap between S1 and S2 have opposing effects on behavioral performance.

To investigate event-file processing, a standard event-file coding task was administered (Kleimaker et al., 2020; Takacs et al., 2020b). The paradigm is shown in Figure 1.

Participants completed the task on a 17-inch CRT computer screen positioned at a distance of approximately 60 cm. During the task, participants were presented 2 consecutive stimuli preceded by a response cue. All were shown in 1 of 3 vertically arrayed boxes in the middle of the screen, each of which measured 2.4 × 0.9 cm. The cue, represented by an arrowhead pointing either left or right, was displayed in the middle box. The following stimulus 1 (S1) and stimulus 2 (S2) both consisted of either a horizontal or a vertical line. Further, S1 and S2 were either red or green and were presented either in the top or the bottom box. Consequently, the features of the stimuli could differ in 3 aspects: orientation (horizontal or vertical), color (red or green), and position (top or bottom). The degree of feature overlap between S1 and S2 varied randomly throughout the task, resulting in trials with full feature overlap (identical S1 and S2), partial feature overlap (1 or 2 features were shared by S1 and S2), and no feature overlap (no features were shared between S1 and S2).

During each trial, first, the cue was presented for 1500 milliseconds. Subsequently, S1 was shown for 500 milliseconds and S2 was displayed until a response was given, or for a maximum of 2000 milliseconds. The presentations were separated by a blank screen shown for 1000 milliseconds after the cue and for 2000 milliseconds after S1. Per trial, the execution of 2 responses (R1 and R2) was required by pressing either the left or right control key of the computer keyboard with the respective index finger. First, participants were instructed to indicate the direction of the arrowhead by pressing the corresponding left or right key. They were asked to not react immediately but to delay their response until S1 appeared. Although carried out simultaneously with the presentation of S1, the type of R1 (control key press with the left or right index finger) was independent of the features of S1. In the event of an erroneous R1, the trial was repeated. On the presentation of S2, participants were asked to immediately press the left key for a horizontal line and the right key for a vertical line, independently of color and position. The consecutive R1 and R2 could entail a response repetition (pressing the same key) or a response alteration (pressing a different key). Participants were told that there would be no systematic pattern in the degree of feature overlap between S1 and S2 or in the relationship between S1 and R1. In the task, people are faster when facing the repetition of stimulus shape and response features (i.e., complete repetitions; e.g., both stimulus shape and the response) or when facing an entirely different combination (i.e., complete alternations). In contrast, people are slower when facing a new combination of the same features (i.e., partial repetitions; e.g., shape but not the response or the response but not the shape). In statistical terms, these binding effects are reflected by a significant interaction of the factors “response” (repetition vs alternation) and “feature overlap” of the S1 and S2 stimuli (Colzato et al., 2006; Petruo et al., 2016). This interaction is hypothesized to be further modulated by MPH vs placebo administration as well as the order of MPH/placebo administration in the cross-over design. The entire task consisted of 384 trials separated by inter-trial intervals varying between 1500 milliseconds and 2000 milliseconds, during which a fixation cross was presented in the middle of the display. Depending on the amount of incorrect R1 responses, the number of trials could increase to 395.

### Statistical Analysis

The statistical analysis for the behavioral data was carried out with SPSS Version 25.0. For each participant, the percentage of correct responses (accuracy) as well as the mean reaction times (RTs) were calculated. To analyze the data, mixed effects 4 × 2 × 2 × 2 ANOVAs with “group” as a between-subject factor and “substance,” “feature overlap,” and “response” as within-subject factors were performed. The factor “group” was based on whether a participant had received the placebo in the first session (and MPH in the second session; placebo-first) or MPH in the first session (and the placebo in the second session; MPH-first). The factors “group” (placebo-first vs MPH-first), “substance” (placebo vs MPH), and “response” (repetition vs alternation) each had 2 levels. The factor “feature overlap” of the S1 and S2 stimuli had 4 levels (no feature overlap, 1 overlapping feature, 2 overlapping features, or overlap of all 3 features). For the descriptive statistics, the mean and SD are given. To account for a lack of sphericity, a Greenhouse-Geisser correction was applied when necessary.

## Results

### Behavioral Data

#### Accuracy

The mixed-effects ANOVA of the percentage of correct responses to the S2-stimulus with “group” as a between-subjects factor and “substance,” “response,” and “feature overlap” as within-subject factors showed a significant main effect for “substance” (*F*_(1,43)_ = 11.39, *P* = .002, ƞ _p_^2^ = .209), with higher accuracy in the MPH session (91.71% ± 4.77) than in the placebo session (89.74 % ± 6.19). Moreover, there was a significant main effect for “feature overlap” (*F*_(3,129)_ = 4.49, *P* = .010, ƞ _p_^2^ = .0.95), with a lower accuracy in the 3 feature overlap trials (89.53% ± 6.19) than in the 2 feature overlap trials (91.29% ± 5.13, *t*(44) = 3.01*, P* = .004, *d* = .449) and in the 1 feature overlap trial (91.18% ± 5.05, *t*(44) = 3.17, *P* = .003, *d* = .472). No other pairwise differences were significant (all *t* < |1.96|, all *P* > .057). Furthermore, the results showed a significant interaction effect for substance × group (*F*_(1,43)_ = 23.92, *P* < .001, ƞ _p_^2^ = .357), response × feature overlap (*F*_(3,129)_ = 80.67, *P* < .001, ƞ _p_^2^ = .652), substance × response × group (*F*_(3,43)_ = 4.16, *P* = .048, ƞ _p_^2^ = .088), and substance × response × feature overlap (*F*_(3, 129)_ = 4.32, *P* = .010, ƞ _p_^2^ = .091).

**Figure 2.**
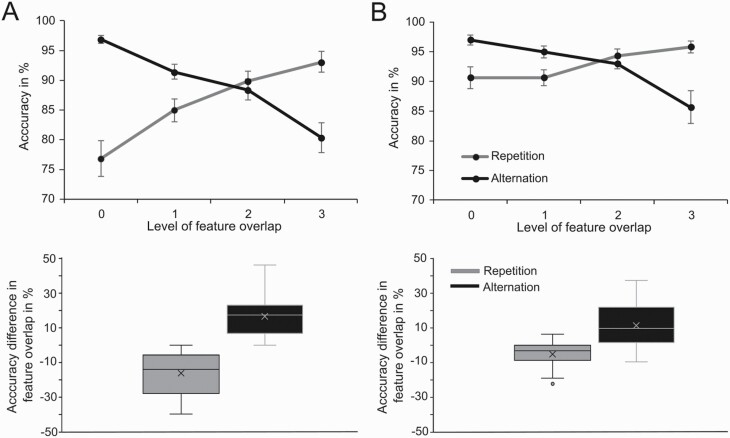
(A) Above: The accuracy (correct responses in %) rates of the placebo-first group in the placebo session is displayed as a function of the level of feature overlap for the repetition trials (black) and for the alternation trials (grey). Below: The difference in accuracy between the zero feature overlap level and the 3 feature overlap level is shown as a function of response (repetition or alternation) for the placebo session of the placebo-first group. (B) Above: The accuracy (correct responses in %) rates of the placebo-first group in the methylphenidate (MPH) session is displayed as a function of the level of feature overlap for the repetition trials (black) and for the alternation trials (grey). Below: The difference in accuracy between the zero feature overlap level and the 3 feature overlap level is shown as a function of response (repetition or alternation) for the MPH session of the placebo-first group. The means and standard errors of the mean are provided.

Importantly, there was a significant 4-way interaction including all experimental factors, that is, group × substance × response × feature overlap (*F*_(3,129)_ = 7.76, *P* < .001, ƞ _p_^2^ = .153). A post-hoc analysis revealed that the interaction substance × response × feature overlap was not significant in the MPH-first group (*F*_(3,66)_ = 0.33, *P* = 0.735, ƞ _p_^2^ = .015) but only in the placebo-first group (*F*_(3,63)_ = 11.89, *P* < .001, ƞ _p_^2^ = .361). Consequently, the analyses described in the following only refer to the placebo-first group. In both the placebo and the MPH session, the accuracy rate increased from the zero feature overlap level to the full feature overlap level for repetition trials (*F*_(3,63)_ = 25.89, *P* < .001, ƞ _p_^2^ = .552 in the placebo session; *F*_(3,63)_ = 7.17, *P* = .002, ƞ _p_^2^ = .254 in the MPH session) and decreased from the zero feature overlap level to the full feature overlap level for alternation trials (*F*_(3,63)_ = 33.20, *P* < .001, ƞ _p_^2^ = .613 in the placebo session; *F*_(3,63)_ = 16.51, *P* < .001, ƞ _p_^2^ = .440 in the MPH session). The increase of accuracy with increasing feature overlap for the repetition trials and the decrease of accuracy with increasing feature overlap for the alternation trials can be seen in Figure 2A for the placebo session and in Figure 2B for the MPH session. This pattern of results generally reflects the binding effects (partial repetition costs and benefits) established by previous studies (Hommel, 2004, 2009; Colzato et al., 2006).

Next, to examine binding effects in more detail, the difference between the accuracy at the zero feature overlap level and at the full feature overlap level was calculated separately for the repetition trials and the alternation trials for both the placebo session and the MPH session. The value pertaining to the full feature overlap level was always deducted from the value pertaining to the zero feature overlap level. Subsequently, these differences were compared with each other. Comparing the estimates of the strength of the partial repetition benefits, the analysis revealed that the increase in accuracy during the repetition trials was significantly larger in the placebo session (−16.13 ± 11.83) than in the MPH session (−5.16 ± 7.54, *t*(21) = −4.01, *P* = .001, *d* = −.854). Furthermore, comparing the estimates of the strength of the partial repetition costs, the decrease in accuracy during the alternation trials was significantly larger in the placebo session (16.56 ± 10.69) than in the MPH session (11.32 ± 12.03, *t*(21) = 2.49, *P* = .021, *d* = .531). Notably, the effect size was shown to be larger for the repetition trials than for the alternation trials: the Cohen’s *d* value of −.854 in the repetition trials can be interpreted as a large effect while the Cohen’s *d* value of .531 can be interpreted as a moderate effect (Cohen, 1988), indicating that the interaction effect is driven by the repetition trials. Therefore, the following analyses examine the repetition trials in more detail.

Comparing the different feature overlap conditions against each other (between the MPH session and the placebo session), there was a significant difference for the zero feature overlap level (*t*_(21)_ = −5.26, *P* < .001, *d* = −1.12), for the 1 feature overlap level (*t*_(21)_ = −3.65, *P* = .001, *d* = −.78), for the 2 feature overlap level (*t*_(21)_ = −3.15, *P* = .005, *d* = −.67), and the full feature overlap level (*t*_(21)_ = −2.19, *P* = .040, *d* = −.47). The effect size for the difference between the MPH session and the placebo session in the repetition trials was the largest in the zero feature overlap level (*d* = −1.12), warranting further post-hoc tests in this regard. Further analyses revealed that the difference in accuracy between the placebo session and the MPH session at the zero feature overlap level (−13.72 ± 12.24) was significantly larger than the difference in accuracy between the 2 sessions at the 1 feature overlap level (−5.68 ± 7.29, *t*_(21)_ = −3.27, *P* = .004, *d* = −.697), the 2 feature overlap level (−4.48 ± 6.73, *t*_(21)_ = −3.63, *P* = .002, *d* = −.775), and the 3 feature overlap level (−2.75 ± 5.89, *t*_(21)_ = −4.01, *P* = .001, *d* = −.854). The difference in the accuracy between the placebo and the MPH session for each other comparison of the different overlap levels did not significantly differ from each other (all t < |1.65|, all *P* > .115). There were no other significant main or interaction effects for accuracy (all *F* < 3.07, all *P* > .087).

#### RTs

The mixed-effects ANOVA of RTs for correct responses revealed a significant interaction effect for response × feature overlap (*F*_(3,129)_ = 46.74, *P* < .001, ƞ _p_^2^ = .521), which was not further modulated by the factor “substance” or “group.” The detailed analysis of the RTs data can be found in the supplementary Material.

## Discussion

In the current study, we examined how modulations of the catecholaminergic system using MPH affects the integration of perception and action while taking into consideration the impact of learning. Prior task experience is of particular relevance because learning processes have been suggested to affect the processing of event files and hence the integration of perception and action (Eberhardt et al., 2017). Specifically, the processes underlying learning and binding have been demonstrated to be distinct from each other (Moeller and Frings, 2017a), and learning experience has been found to modulate binding processes (Moeller and Frings, 2017b), for example, with strongly overlearned S-R associations impeding the new integration of stimulus features and responses. Moreover, the effects of MPH on various cognitive control processes have been shown to depend on prior learning or task experience (Bensmann et al., 2019; Mückschel et al., 2020a, 2020b). The data show that the effects of 0.25 mg/kg immediate-release MPH on the strength of perception-action integration was modulated by prior task experience. In the experiment conducted in the present study, binding effects were reflected by a significant interaction of the factors “feature overlap” and “response.” Importantly, this interaction was further modulated by the factors “substance” (MPH vs placebo) and “group” in the accuracy data. No such modulations were seen for the RT data, the reason for which remains unclear. Consequently, we focus on the accuracy data in the following discussion.

The factor “group” refers to the order in which MPH and the placebo were administered in the cross-over study design. While it is possible that different factors associated with the study design, that is, conducting the assessments at 2 different points in time, may have influenced the obtained results, prior evidence from neuropsychopharmacological studies on the same compound strongly suggests that learning effects play a role when modulating the catecholaminergic system. Compared with the placebo, MPH affected perception-action integration only when it was administered in the second session (i.e., in the placebo-first group). No MPH effects on event file binding (i.e., the interaction “feature overlap × response”) were evident when MPH was administered in the first session. Thus, the results show that prior learning experience modulates MPH-induced effects of catecholaminergic system activity during perception-action integration. Without prior learning experience, MPH did not modulate event file binding effects (compared with the placebo). When considering possible neurobiological mechanisms that may underlie these effects, it is important to take into account the magnitude of the MPH effects after prior learning experience. The event file binding effect is maximal between the zero feature overlap and the full feature overlap condition. Interestingly, the magnitude of the performance difference between these conditions was smaller after MPH administration than after placebo administration. Therefore, the data reveal that, compared with the placebo, the effects of event file binding become smaller after administering 0.25 mg/kg MPH. This was the case for trials in which the response had to be alternated as well as for trials where the same response had to be executed. However, the modulatory effects were larger in trials were the motor response was repeated (*d* = |.854|) compared with the trials where the response was alternated (*d* = |.531|). Within the repetition trials, differences between MPH and placebo were strongest in the zero feature overlap condition and decreased in magnitude with increasing feature overlap between S1 and S2 stimuli (cf. Methods section). In repetition trials, the zero feature overlap condition is the most demanding one since the same response has to be selected on the basis of completely different S2 stimulus input (cf. Methods section). MPH administration was associated with a better performance in the zero feature overlap condition as well as in all other conditions.

Previous data have suggested that the effects of MPH are modulated by prior task experience and that MPH effects and learning or task experience-related modulations seem to tap into highly similar neural mechanisms (Bensmann et al., 2019). In particular, it has been suggested that the common neural mechanisms modulated by learning and MPH are related to gain control (Bensmann et al., 2019). High neural gain facilitates response selection processes (Aston-Jones and Cohen, 2005; Nieuwenhuis et al., 2005; Chmielewski et al., 2017; Mückschel et al., 2017; Adelhöfer et al., 2019), which may explain the superior task performance (i.e., higher rate of correct responses). High neural gain enables high performance under conditions where the event file has to be reconfigured to respond correctly. Moreover, gain control mechanisms are closely related to processes induced by learning and plasticity (Dosher and Lu, 1998; Gold et al., 1999), given that learning also enhances the neural signal-to-noise ratio in neural circuits. Therefore, gain control principles are relevant in explaining the effect of prior task experience. Likely, prior task experience in the current study induced learning and plasticity mechanisms and may thereby have modulated gain control. Interestingly, MPH was not able to alter event file coding when administered in the first session of the cross-over study design, thereby suggesting that the administered dosage of MPH was not able to sufficiently modulate gain control processes during perception-action integration. However, when gain control principles during perception-action integration were pre-modulated by prior learning/task experience, MPH was able to modulate event file coding. The interactive modulatory effect of task experience and the modulation of the catecholaminergic system by MPH is a result of both factors being based on a common underlying mechanism (gain control).

The possible neurobiological mechanism outlined above has some similarities with the Yerkes-Dodson principle. According to this principle, there is an inverse U-shaped relationship between catecholaminergic concentration and performance (Aston-Jones and Cohen, 2005; Cools and D’Esposito, 2011). One might assume that the administration of 0.25 mg/kg MPH is not enough to alter the catecholaminergic concentration sufficiently to shift the performance in the task to an “optimum range” for the cognitive processes under investigation. However, when prior learning has occurred, the MPH-induced change in catecholaminergic activity may be sufficient to bring about this shift, seeing as it may add to the effects of prior task experience and plasticity. In this respect, it should not be disregarded that such mechanisms may also underlie the effects observed in the present study. A limitation of the study is that no other (higher) MPH dosages were administered, and it might prove worthwhile for future studies to compare the effect of different MPH dosages on the efficacy of event file binding. This is particularly the case because shifting performance level beyond an optimal point (e.g., by increasing MPH doses) can then alter the inter-related of prior learning experience and catecholaminergic modulation. However, in this regard, it should be taken into consideration that such a Yerkes-Dodson relationship was originally described and validated for working memory processes and the stability of representations in working memory (Kentros et al., 2004; Seamans and Yang, 2004; Robbins and Arnsten, 2009; Hamilton et al., 2010; Arnsten, 2011; Cools and D’Esposito, 2011), which is also relevant in the context of event file coding processes (Hommel, 2004, 2009; Takacs et al., 2020a). The behavioral results provided by the experimental paradigm used in this study can only occur when (1) stimulus features and the response were bound in the first part of a trial in the experiment, and (2) when this binding was retrieved in the second part. Therefore, the behavioral results may not only reflect binding but also the retrieval of the binding, or both processes. However, an increased strength of bindings within the event files entails worse task performance in conditions where previously established bindings between stimuli and responses have to be reconfigured (Hommel, 2009; Takacs et al., 2020a), such as in the zero feature overlap condition in response repetition trials (Colzato et al., 2006). Especially in this condition, the combined effect of prior task experience and MPH led to a high task performance. Accordingly, it could be concluded that the stability of working memory representations and bindings per se may only play a minor role, and the data pattern can best be explained by a gain control–based mechanism affecting response selection underlying the interactive effects of learning/task experience and catecholaminergic activity during perception-action integration. Nevertheless, future studies could investigate the possibility that MPH primarily affects the retrieval of S-R bindings and not the strength of S-R bindings in more detail using paradigms allowing to dissociate binding from response selection and retrieval effects (Frings et al., 2020). In this regard, it is worth mentioning that such paradigms have already been developed (Moeller and Frings, 2017a) and reveal evidence that it is important to distinguish more short-lived binding effects (examined in this study) from learning of longer-lasting stimulus response bindings. A question for future studies is hence whether the observed effects do generalize to longer-lasting S-R bindings or are confined to short-lived binding effects. Considering the main finding of “prior task experience” modulating the effect of MPH, it is interesting that previous work has shown that “overlearned” S-R associations cannot be integrated in novel, to-be established S-R associations (Moeller and Frings, 2017b). Regarding this, it is possible that the modulatory effects of MPH on S-R bindings may also change depending on the degree of learning/prior task experience. This will also be important from another perspective. The interactive pattern of results shows that learning/task experience effects cannot be disentangled from MPH effects. This is why both factors are interacting. If learning/task experience would not share a common ground, additive effects would emerge. However, when varying the amount/degree of prior task experience independently from MPH dose, one can delineate which of these 2 factors has a stronger effect on event file coding.

An interesting, unexpected result is the finding that the interactive effects of learning/task experience and MPH intake were larger in trials where the motor response was repeated compared with the trial where the response was alternated. This has important implications for the cognitive framework motivating the current study. In its present form, theory of event coding states that partial repetition costs and benefits are both consequences of the same mechanism binding perception and action (Hommel, 2004, 2009). The differences in MPH effects between response repetition and alternation trials, however, suggest that the neurobiological underpinnings of binding mechanisms differ between these trial types, which gives rise to the assumption that there is no unitary binding mechanism in event files. Furthermore, since MPH is the recommended first-line treatment in ADHD (American Academy of Pediatrics, 2011; Childress and Sallee, 2014; Mattingly et al., 2017), which is also accompanied by deficits in perception-action integration, the current findings have strong implications for the efficacy of MPH treatment in ADHD, which may be investigated in future studies. Based on the key role catecholamines play in the response to stress (Goldstein, 1995), it might furthermore prove worthwhile to examine binding effects in relation to stress levels in future investigations.

In summary, the study shows how the integration of perception and action is modulated by the catecholaminergic system. The results show that increasing catecholaminergic concentrations using MPH leads to better performance when perception-action bindings have to be reconfigured. Yet, this was only the case when participants had already acquired experience with the task—that is, when learning effects were already evident. The data suggest that there is a gain control–based mechanism underlying the interactive effects of learning/task experience and catecholaminergic activity during perception-action integration.
